# Effect of Education for Hypertensive Patients with Correctly Performed Self-Blood Pressure Monitoring (SBPM)

**DOI:** 10.3390/pharmacy7030075

**Published:** 2019-06-27

**Authors:** Justyna Dymek, Anna Gołda, Wioletta Polak, Bartosz Lisowski, Agnieszka Skowron

**Affiliations:** 1 Department of Social Pharmacy, Faculty of Pharmacy, Jagiellonian University Medical College Medyczna, 9 Street, 30-688 Kraków, Poland; 2 Department of Biophysics, Jagiellonian University Medical College, św. Łazarza 16 Street, 31-530 Kraków, Poland

**Keywords:** patient education, knowledge, skills, hypertension, self-monitoring

## Abstract

Introduction: The study objective was to assess the impact of pharmacist-led education on the patient’s knowledge and skills on SBPM (self-blood pressure monitoring). Methods: Patient knowledge was assessed using tests and patients’ skills were based on a checklist (20 parameters) completed by the researcher based on the SBPM video records. Patients taking pressure measurements were filmed for 20 days and after 6 months. After the first 10 days, patients were educated about the correct SBPM procedure. Knowledge tests were repeated three times (before/after/6 months after education). Results: All patients’ knowledge and skills in the field of SBPM were improved after education. After the education, patients scored an average of 9 out of 10 points in the knowledge test (increasing an average of five points after education), six months later—an average increase of 7.36 points. Patient skills after training were rated at 17.4 out of 20 points on average (increase by an average of 5.14 points after education), six months later, there was an average of 16.23 points. Conclusions: The study showed an increase in patients' knowledge and skills in the field of SBPM after training.

## 1. Introduction

Scientific publications and guidelines of scientific associations indicate the significant role of self-blood pressure measurements (SBPM) in the process of arterial hypertension therapy monitoring and for the therapeutic decision-making [1,2,3,4,5,6]. It is recommended that a home blood pressure monitor (HBPM) is a routine activity in arterial hypertension therapy [7,8]. A self-measurement actively involves the patient in the arterial hypertension control process, increases their awareness of the disease, facilitates detection of situations with negative impact on the value of blood pressure and increases motivation for therapy monitoring. Self-measurement is the preferred form of blood pressure control, as compared with Ambulatory Blood Pressure Monitoring [9]. All those factors contribute to reduction of the frequency of failing to observe therapeutic recommendations, which have considerable impact on the disease control level [10,11,12,13,14]. 

SBPM constitutes an indispensable assistance in the control of the health state of a patient only when the measurement is correctly conducted and it complies with the recommendations/guidelines of scientific associations, since only then the obtained results are reliable [1,6,8,15,16,17].

Unfortunately, the knowledge as well as skills of patients in this field are insufficient [1,2,6,8,15,16,17,18,19,20]. Patients fail to carry out regular arterial blood pressure measurements and do not use recommended pressure gauges [6]. The most common errors made during the SBPM procedure include attaining incorrect body position, improper manner of resting the arm against a table top, incorrectly put on cuff, lack or too brief rest prior to the measurement or clenching the palm during measurement [6]. Prior to commencing self-blood pressure measurements, patients should be trained in this field. Enhancement of the knowledge of patients on blood pressure measurements motivates them to undertake regular measurements [13]. 

## 2. Materials and Methods

The study objective was to assess the impact of training process on the change of the knowledge and skills levels of patients with arterial hypertension in the field of self-blood pressure measurement using semi-automatic pressure gauge. Moreover, the usability of the used methods and education tools was also assessed. 

The study was performed in the facilities (general access drugstores and in a healthcare facility) described in the article of Dymek et al. [6]. The methodology described in the article of Dymek et al. constitutes the first part of the presented study, including patients covered by the study. The patients expressed consent to participate in the study and to use audiovisual means recording the patients’ measurements. 

Within the study, patients performed self-blood pressure measurements using the provided, individually to each patient, arm monitors, which were semi-automatic with built-in memory. The choice of a semi-automatic device, instead of a fully automatic device, allowed patients to master the ability to independently select the pressure value to which the cuff should be pumped. This type of pressure device is cheaper than fully automatic ones and therefore more often used among Polish patients.

**Figure 1:**
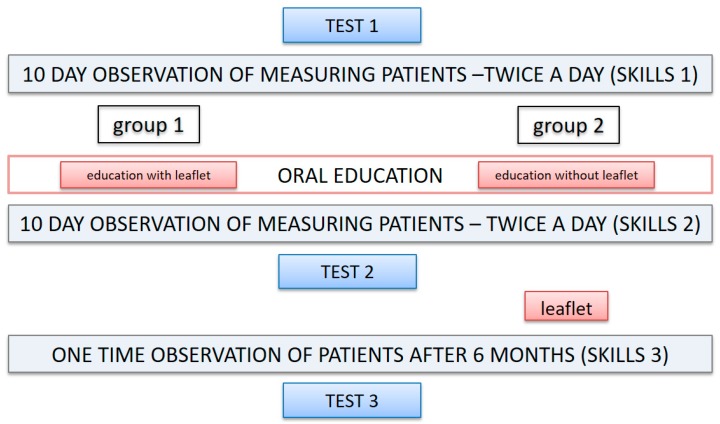
Time schedule of the study.

The measurements were performed twice a day for 20 consecutive work days in the presence of a researcher. The measurement was recorded using audiovisual means. After the first 10 days, each patient had individual educational meeting with researcher concerning the principles of proper blood pressure measurement (Figure 1). The topics of each training session were identical for each study participant. Differences in the answers provided concerned individual issues, e.g., value of cuff inflation or selection of arm for the measurement. The correct course of blood measurement was discussed, including: the role of SBP.M.; suitable conditions prevailing in the room during measurement, preparation for the measurement (e.g., rest, proper cuff attachment), proper performance of the measurement, including arm selected individually for the measurement and the value of cuff inflation, recording and interpretation of the results. In addition, every patient was provided with information on the most frequent errors made during measurement during the training. This information originated from analysis of the first 10 days of measurements. Results of this analysis were presented in the article of Dymek et al. [6].

Prior to the process of education, patients were assigned at random to two groups (group 1, group 2), in accordance with the simple randomization principles using dependent randomization. During training, patients in group 1 were provided with individualized leaflets containing information provided in the course of the meeting. Patients were able to use the leaflet during subsequent measurements. Patient education in group 2 was not supported with written information during an educational meeting. Patients in this group received an educational leaflet after completing TEST 2 (Figure 1). After 10 subsequent measurements, the participants were provided with a pressure monitor and self-testing logbook for the performance of regular measurements at home for the period of the next 6 months. 

Assessment of the patient knowledge in the field of self-blood pressure measurement (SBPM) was performed based on the results of knowledge test results. The test contained 10 questions, and was identical for all study participants at any stage of the study (issues from the questions are included in Table 1). The test was solved on three occasions: at the onset of the study (TEST 1), after the training, 20 days from the onset of the study (TEST 2) and at least 6 months after the training (TEST 3).

Patients’ skills in the field of SBPM performance were assessed based on analysis of video recordings of measurements [6]. The method of performing 20 parameters influencing the correctness of the performed measurement of arterial blood pressure, thus influencing the authenticity and reliability of the obtained result were assessed (the assessed parameters are included in Table 2). Each correctly performed parameter was awarded with two points. The assessed parameters, as well as all issues discussed in the knowledge tests were selected based on the guidelines of scientific associations [1,2,4,8].

The presented study was a qualitative study. Because the study was time-consuming, a small number of patients was included in the study. For example, the analysis of films by two independent researchers lasted over 800 hours in total. Therefore, the quantitative analysis was conducted as an additional analysis of the obtained data.

To confirm the normal distribution of the data, the Ryan-Bozer test was used. The differences between data with normal distribution dependent on quantitative variables were verified with an ANOVA test with repeated measurements. When the nature of the variables did not allow the ANOVA analysis, the Kruskal-Wallis test was used.

Ethical approval for the study was obtained from the local ethics committee. All patients provided written for participation in the study, for video recording their measurements and to process their data.

## 3. Results

The result analysis covers data originating from 14 patients diagnosed with arterial hypertension, who completed at least two first stages of the three-stage study. The visit after 6 months was attended by 13 patients. The mean patient age was 59 (29–86 years, median 60.5 years). Over a half of the participants (57.14%) consisted of persons with secondary education, those with university and primary education made up 21.43% of the total. The study included two men. Patients suffered from arterial hypertension for the period of 6 months to 32 years (6).

Patient knowledge was assessed based on a three repetitions of test containing 10 questions. After the training, the knowledge on SBPM increased in all patients by a mean of 5 points (TEST 2 vs TEST 1 p < 0.0001). In this test, patients scored a mean of 9 points out of 10. In TEST 3, the results were reduced relative to TEST 2 (p < 0.02), by an average of 1 point. Finally, the TEST 3 results remained significantly higher relative to those obtained in TEST 1 (p < 001). In TEST 3, patients scored an average of 7.36 points. The difference in the percentage of persons responding correctly to individual test questions is presented in Table 1. 

The greatest increase (by over 70%) of the percentage of correct answers in TEST 2 as compared with TEST 1 concerns the question on the time interval between a hearty meal and the SBPM and on the cuff inflation value (Table 1).

In TEST 3, as compared with TEST 2, a decrease in the percentage of correct answers to seven test questions occurred. The greatest decrease of the percentage of correct answers concerns questions on the selection of arm for the measurement, time lapse between administration of medicines before the measurement and the time interval between measurements (decrease by over 20%).

The final increase in the knowledge, i.e., comparison of TEST 3 and TEST 1, concerns all questions and it amounts to 4.4% in the case of question on the time interval between medicine administration and measurement to 64.3% in the question on the factors influencing result reliability. Details on the increase of knowledge on the individual test questions are presented in Table 1. 

The results failed to reject normality with the Ryan-Joiner test at a 0.01 significance level. Assuming normality of distribution, the t-Student test (paired t-test) for the dependent samples has demonstrated that differences in the percentage of correct answers between TEST 2/TEST 1; TEST 3/TEST 2; TEST 3/TEST 1 are statistically significant (p = 0.05). Results were verified with a non-parametric Kruskal-Wallis test, which demonstrated that medians of differences between the percentage of correct answers in the appropriate combinations are significantly different (p = 0.05).

By comparing the increase of knowledge in SBPM in both analyzed groups (Table 3), difference testing was performed using the ANOVA with repeatable measurements. Based on this analysis, an absence of significant differences between three test results within group 1 versus group 2 was found.

Assessment of patients’ skills during self-blood pressure measurement was performed on the basis of analysis of 1064 video recordings of measurements performed by 14 patients. In all patients, an increase of skill in SBPM performance was observed after the training, both in the second as well as in the third study stage. Measurements performed by patients after training were rated at 17.34 points on average (max. 20). This means an average increase of 5.14 points relative to measurements prior to training (in group 1 patients by 5.46 and in group 2 patients by 4.81). In both groups, a decrease in the skill level was observed after 6 months from training. Results of skill analysis after this period demonstrated that patients scored a lower number of points on average than after the training. However, this result was still higher than the result obtained prior to training (average increase by 4.29 points in group 1 and by 3.89 points in group 2). Based on the ANOVA with repeatable measurements it can be stated that patients of group 1 and group 2 do not differ statistically (p = 0.65) within the range of results determining the ability to perform self-blood pressure measurement prior and after training and after a 6-month period after training. The increase in skills of both patient groups is presented in Table 3.

After the training, the greatest increase of skills expressed by the number of points scored per measurement concerned assuming the correct body posture during measurement, patients sat facing the table more frequently (increase by 63%), they kept back upright (increase by over 58%) and rested the back against the chair’s backrest (increase by over 83%) (Table 2). 

Results presenting increase of skills were verified with non-parametric Kruskal-Wallis test, which has demonstrated that medians of differences between the percentage of correct answers in the appropriate combinations (SKILLS 2/SKILLS 1, SKILLS 3/SKILLS 2, SKILLS 3/SKILLS 1) are significantly different (p = 0.05). 

Mean pressure values were compared before and after the training for each patient individually. For comparison of pressure values, results obtained by patients within 10 days before and 10 days after education were used. This analysis does not take into account the results of measurements made after 6 months, because their value, apart from the method of pressure measurement itself, could have been influenced by changes in lifestyle and pharmacotherapy during the next 6 months.

In two patients, mean systolic pressure and diastolic received during 10 days of measurements after education was higher than before education (an increase of 7% on the initial value of Systolic Blood Pressure (SBP) and Diastolic Blood Pressure (DBP) on average). In 10 patients, there was a decrease in mean systolic and or diastolic pressure in the measurements performed after the educational meeting. The most significant reduction of the pressure values is a 10% decrease relative to the initial SBP and 17% compared to the initial DBP.

## 4. Discussion 

In the presented study, a pharmacist was the educator, which is part of the role of a modern pharmacist [21,22,23,24]. Education of patients was performed in a verbal form; in addition to that, written, individualized educational materials were prepared for patients. The education process was planned and implemented in compliance with the forms of health education forms described in the global literature [21,25,26,27,28,29,30], as well as in accordance with preferences of patients themselves, who value training in verbal form [30,31]. Meetings were organized in a place ensuring privacy and discretion, which constitutes an important factor for patients [31,32].

Education of all patients was performed by one educator. Thus, the issue arising in scientific publications was eliminated, consisting in the influence of differences in the level of substantive knowledge, communication competences or styles of providing information by various educators [33,34]. In addition, the educator’s role also consisted in observing the SBPM performance by patients as well as the interviewer conducting knowledge tests with patients. This enabled explanation of questions included in the knowledge tests, without educating elements, which vastly increased the level of question understanding, and thus the reliability of the answers provided [35].

Education of each patient was expanded with analysis of knowledge test results as well as analysis of video recordings of SBPM performance. This enabled adjustment of educational (verbal and written) content to the individual needs of each patient. The available literature data indicate that individualized provision of information during training greatly impacts the efficiency of an education process [36,37,38,39,40,41]. Moreover, as indicated by the study of Svavarsdóttir and Borgsteede [30,32] individualization of information is, according to patients, the most important element influencing their positive assessment of a training.

Following the procedure described in the literature, in order to assess the efficiency of the educational activities undertaken, tools used by the educator to assess the knowledge and skills of patients prior to the training were also used after the education [42,43,44].

An undisputed advantage of the conducted education was the use of semi-automatic devices, provided to each patient. Blood pressure measuring devices, according to the guidelines, were validated according to internationally accepted protocols [45]. The devices enabled the educator to demonstrate the manner of properly conducted SBP.M.; which greatly increases efficiency of the education [25]. Moreover, the selection of a semi-automatic device, instead of a fully automatic device, enabled mastering the ability to individually select the pressure value to which the cuff should be inflated. 

As presented by the study, verbal education conducted in all patients covered by the study constitutes an efficient training method. This remains in line with the study results of Axtell et al., which confirmed the high efficiency of direct trainings; furthermore, it indicated a considerably higher knowledge level and patient skills using this educational technique as compared with other education forms [44]. However, contrary to the study of Dawes et al. [10] and the publication of Johnson et al. [40], this study has demonstrated that enriching verbal education with educational leaflet does not contribute to enhancement of the effect of verbal education. A comparison of the level of knowledge and skills of patients from group 1 and group 2 demonstrated the absence of statistically significant differences within the results assessing the knowledge and skill of SBPM procedure prior and 2 weeks after training, which is within a brief period of time after verbal education.

Following the data analysis from the present study and the mentioned publications, we can assume that the influence of educational leaflet on the increase of knowledge level is significant in the situation when the educator uses only this educational tool, completely replacing verbal education. This is confirmed by the study by Axtell et al. as well as in the study by Dawes et al. [10,44]. The use of leaflets for a longer period of time following verbal education further contributes to maintaining and strengthening the effect of verbal education [25].

Patients’ education increased the ability to perform self-blood pressure measurement by the patients, which became apparent by the reduction of the frequency of errors made during measurement. As a result of education, the percentage of correctly performed parameters increased from 61% to 86.7%. It appears that the presented study is the first one providing such a complex analysis of the impact of education on the ability to correctly perform 20 different elements of SBPM. The analyzed, available studies typically analyze the pressure measurement method, including errors during the performance of such a measurement and regularity at which it is performed. Those studies typically assess in detail one selected aspect, without examining the influence of education on the change of behavior and patient skills [6,12,46,47]. 

Following the assumptions of studies on health education of patients with chronic diseases, the present study also examined the durability of effects of the conducted education after 6 months [15,48]. As shown by the results of this study, the level of knowledge and skills acquired during educational meetings decreases with time. This phenomenon has been confirmed by the results of studies performed by Adams and Axtell et al., who believe that a singular education is ineffective and indicate the need for periodic repetition of the education process [44,49]. In the study of Svavarsdóttir et al., the patients themselves expressed the need to repeat education after a certain amount of time passed [32]. One of the solutions to this problem, presented by Hunt et al., is providing patients with educational materials via e-mail, which, thanks to regular delivery, systematically motivate patients to extend their knowledge or to maintain correct health behavior [50].

## 5. Conclusions

The study has demonstrated an increase of knowledge under the impact of education conducted by the pharmacist in all patients who participated in the study. Moreover, the conducted training caused an increase in the skills of self-blood pressure measurement, which translates into the reduced frequency of errors made by patients during the measurement. The knowledge and skills levels acquired during educational meetings decrease over time. It is necessary to repeat the education process. According to our results, it could be valuable to re-educate patients at least every six months.

## Figures and Tables

**Table 1:** Percent of patients who give a correct answer and comparison of patients’ knowledge about self-blood pressure monitoring (SBPM) before (TEST 1) and after education (TEST 2) and 6 months after education (TEST 3).

TEST QUESTION	TEST 2 % Patients Who Give a Correct Answer (n), N = 14	Difference Between TEST 2 and TEST 1	TEST 3 % Patients Who Give a Correct Answer (n) N = 13	Difference Between TEST 3 and TEST 2	Difference Between TEST 3 and TEST 1
Arm selection for measurements	78.57 (11)	50.00	53.85 (7)	−24.72	25.28
Requested medicine administration time lapse prior to SMBP	85.71 (12)	28.57	61.54 (8)	−24.17	4.40
Recommended frequency of daily measurement	64.29 (9)	50.00	69.23 (9)	4.94	54.94
Time lapse between two consecutive measurements	100.00 (14)	64.29	76.92 (10)	−23.08	41.21
The value for inflating the cuff	92.86 (13)	71.43	76.92 (10)	−15.94	55.49
Hearty meal – SMBP minimum interval	92.86 (13)	78.57	76.92 (10)	−15.94	62.63
Coffee – SMBP minimum interval	92.86 (13)	64.29	84.62 (11)	−8.24	56.05
Pressure indicating hypertension	100.00 (14)	21.43	92.31 (12)	−7.69	13.74
Body posture during measurement	100.00 (14)	14.29	100.00 (13)	0.00	14.29
Factors impacting readings	92.86 (13)	57.15	100.00 (13)	7.14	64.29

Ratio of correct answers given by the subjects after and before the intervention and 6 months later. Data were tested for normality (Ryan-Joiner Test, failed to reject normality with p = 0.01) and differences between the columns were found to be statistically significant (paired t-test, p < 0.05). Due to small sample size results were verified with Kruskal-Wallis test, which confirmed the result (p < 0.05).

**Table 2:** Percent of patients who correctly performed the parameter and comparison of patients’ measurement skills before education (SKILLS 1), after education (SKILLS 2) and 6 months after education (SKILLS 3).

ASSESSED PARAMETER	SKLILLS 2 % Patients Who Correctly Performed the Parameter	DIFFERENCE BETWEEN SKILLS 2 and SKILLS 1	SKILLS 3 % Patients Who Correctly Performed the Parameter	DIFFERENCE BETWEEN SKILLS 3 and SKILLS 2	DIFFERENCE BETWEEN SKILLS 3 and SKILLS 1
The distance from the table	50.94	49.96	34.62	−16.32	33.64
Rest prior to SMBP	50.38	31.29	50.00	−0.38	30.91
Back against the chair	86.23	82.69	50.00	−36.23	46.46
Arm rested properly	59.43	−1.20	65.38	5.95	4.75
Hand position (up)	75.66	31.57	65.38	−10.28	21.29
No comments on the cuff setting (height, direction)	87.74	66.09	69.23	−18.51	47.58
None excessive activities	85.85	6.72	88.46	2.61	9.33
Air tube on the elbow joint inner side	98.11	27.64	92.31	−5.80	21.84
No hand/arm movement (with the cuff)	73.21	6.48	92.31	19.10	25.58
Air tube in the middle of the elbow joint	97.55	36.13	92.31	−5.24	30.89
Sitting straight (upright posture)	90.75	57.88	92.31	1.56	59.44
Hand open	96.04	26.16	96.15	0.11	26.27
Selected arm rested free on a table	100.00	0.00	100.00	0.00	0.00
Sitting posture	100.00	0.00	100.00	0.00	0.00
No legs movement	97.74	−1.28	100.00	2.26	0.98
SMBP reading recorded in the diary	97.55	−1.07	100.00	2.45	1.38
No conversations	99.62	8.08	100.00	0.38	8.46
Successful at the first attempt	93.02	3.45	100.00	6.98	10.43
Tight-sleeved clothing removed from the arm	98.11	18.19	100.00	1.89	20.08
Facing the table	99.62	63.40	100.00	0.38	63.78

Ratio of correctly performed subsequent steps of blood pressure test after and before the intervention and 6 months later. Since data in column 6ma/a were not normally distributed (Ryan-Joiner Test, p = 0.01), a Kruskal-Wallis test was used (p < 0.05) to find that the differences between all the columns were statistically significant (p < 0.05).

**Table 3:** Comparison of the knowledge and skills level between groups and study stage.

		Group 1	Group 2
**Number of Points (Mean)**			
TEST 1	max 10	4.29	3.71
TEST 2		8.71	9.29
TEST 3		7.67	8.14
SKILLS 1	max 20	12.00	12.40
SKILLS 2		17.46	17.21
SKILLS 3		16.29	16.29
**Mean Difference between Results**			
TEST 2 / TEST 1	max 10	+4.42	+5.58
TEST 3 / TEST 2		−1.04	−1.15
TEST 3 / TEST 1		+3.38	+4.43
SKILLS 2 / SKILLS 1	max 20	+5.46	+4.81
SKILLS 3 / SKILLS 2		−1.17	−0.92
SKILLS 3 / SKILLS 1		+4.29	+3.89

p = 0.65 (Anova) group 1 versus group 2.
